# Kinetics and Activation Strategies in Toehold-Mediated and Toehold-Free DNA Strand Displacement

**DOI:** 10.3390/bios15100683

**Published:** 2025-10-09

**Authors:** Yuqin Wu, Mingguang Jin, Cuizheng Peng, Guan Alex Wang, Feng Li

**Affiliations:** Key Laboratory of Green Chemistry and Technology of Ministry of Education, College of Chemistry, Sichuan University, Chengdu 610064, China

**Keywords:** dynamic DNA nanotechnology, DNA strand displacement, toehold, strand displacement kinetics

## Abstract

Nucleic acid strand displacement reactions (SDRs) are fundamental building blocks of dynamic DNA nanotechnology. A detailed understanding of their kinetics is crucial for designing efficient sequences and regulating reaction networks with applications in biosensing, synthetic biology, biocomputing, and medical diagnostics. Since the development of toehold-mediated strand displacement, researchers have devised many strategies to adjust reaction kinetics. These efforts have expanded the available tools in DNA nanotechnology. This review summarizes the basic principles and recent advances in activation strategies, emphasizing the role of strand proximity as a central driving force. Proximity-based approaches include toehold docking, associative toeholds, remote toeholds, and allosteric designs, as well as strategies that operate without explicit toehold motifs. These methods enable flexible and scalable construction of DNA reaction networks. We further discuss how combining different activation and kinetic control approaches gives rise to dynamic networks with complex and dissipative behaviors, providing new directions for DNA-based nanotechnology.

## 1. Introduction

Hybridization between complementary nucleic acid strands is a fundamental interaction underlying many biological processes. Hybridization follows the Watson–Crick base-pairing rule and is one of the most programmable and predictable molecular interactions, making it widely used in synthetic biological systems [1,2,3,4,5]. Many theoretical and experimental studies have explored hybridization-based technologies, with applications in nanotechnology, biocomputing, biosensing, and medical diagnostics [6,7,8,9,10].

DNA nanotechnology, founded by Nadrian C. Seeman in the 1980s, is one of the most prosperous research fields relying dominantly on nucleic acid hybridization [11]. While DNA nanotechnology emphasized constructing at the beginning of its birth, increasing attention has been made to understand the kinetics of DNA hybridization and to develop new types of hybridization reactions. In 2000, Yurke et al. reported a toehold-mediated strand displacement (TMSD) design, which later became the foundation of what is now called dynamic DNA nanotechnology [12]. In a typical TMSD reaction, hybridization between complementary strands does not begin randomly along the full sequence. An incumbent strand that is prehybridized to one of the complementary strands deprives the duplex region of hybridization capability. Rather, hybridization can only be originated from a restricted colliding window called a toehold domain. The kinetics of TMSD reactions appear to be much faster than strand-exchange reactions without toehold domains. Because of its simplicity and controllability, TMSD has evolved as one of the most widely used building blocks in dynamic DNA nanotechnology [13].

From a kinetic perspective, TMSD is a highly efficient reaction with a rate constant reaching up to ~10^6^ M^−1^s^−1^ [3,14]. Nevertheless, it is often necessary to control the rates of strand displacement to meet certain functionality. A straightforward idea is to manipulate the length of the toehold domain, which offers a coarse-grained tuning capability with near 10-fold increases in displacement rate by increasing one nucleotide in the toehold domain. A maximal rate can often be reached by extending the toehold domain to 7 nt or 8 nt. To further achieve the fine-grained tuning of displacement kinetics, orthogonal tuning strategies are required; this formulates a major driving force for developing new strand displacement reactions and kinetic tuning strategies. To achieve more complex designs in dynamic DNA nanotechnology, it is also critical to develop various activation or deactivation strategies that can switch on or off strand displacement events on demand. Because of its importance, several reviews have previously been reported with focuses on the functional roles of toehold design and tuning strategies for constructing nanoscale machineries or biosensors. This review focuses on the kinetic aspects of TMSD and related displacement strategies, emphasizing their importance in dynamic DNA nanotechnology.

## 2. Proximity-Inducing Nucleic Acid Strand Displacement

All strand displacement reactions can be understood from two critical perspectives: (i) the thermodynamic driving forces that stabilize the final strand-exchanged complexes, and (ii) the kinetic mechanisms that facilitate strand invasion and minimize undesired intermediate or trapped states. Spontaneous displacement occurs when the product is energetically favored, and efficient exchange requires reducing initiation barriers, often via toehold-mediated recognition. Below we outline the theoretical basis of spontaneous strand displacement and summarize its common reaction topologies.

### 2.1. Kinetic Foundation of Strand Displacement Reaction

**Figure 1:**
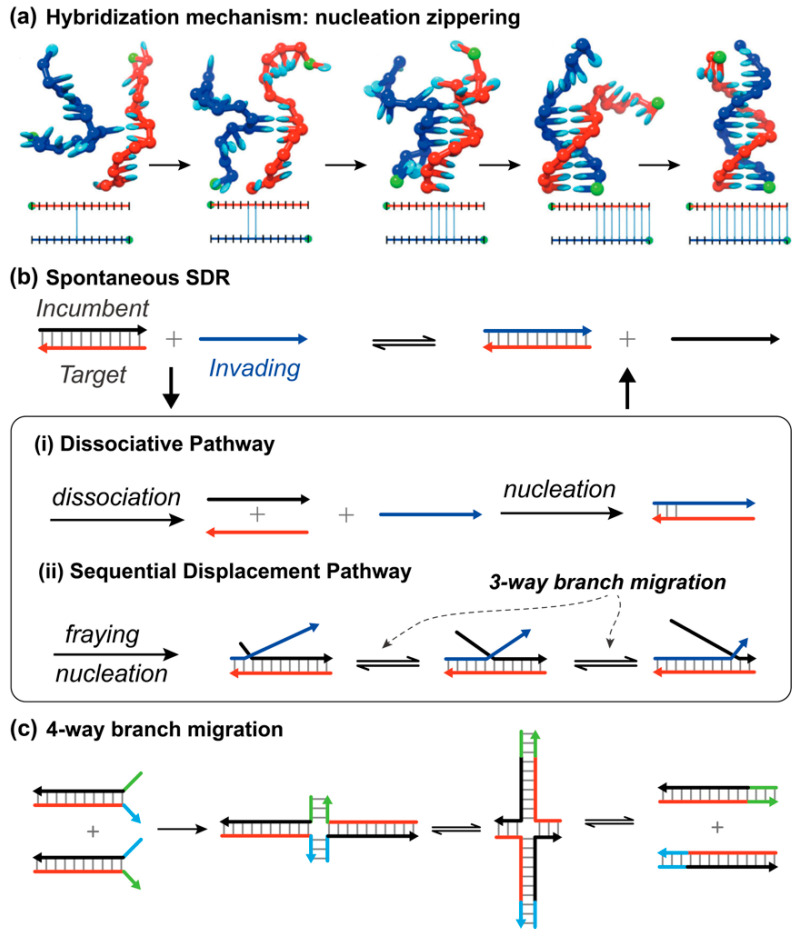
(**a**) Schematic depictions of the hybridization process, where the arrowhead indicates the 3′ end of the DNA strand. Reprinted with permission from ref [15], copyright 2013, Oxford University Press; (**b**) spontaneous strand displacement via dissociative and sequential displacement pathways in ref [17]; and (**c**) the four-way branch migration.

When complementary target strand T and invading strand I come into close proximity, hybridization reaction occurs spontaneously under physiological conditions. It is believed the hybridization reaction undergoes a two-step process where several consecutive base pairs, or so-called nuclei, are firstly formed, then a rapid zippering step proceeds to produce final duplex complexes (Figure 1a) [15]. Since a successful nucleotide requires the strands diffusing to favorable positions for base-pairing and cross-stacking, the nucleation is regarded as the rate-limiting step. Recent reviews by Ashwood and Tokmakoff provide a more detailed discussion of strand hybridization kinetics [16]. In a spontaneous SDR, the strand T is prehybridized by an incumbent strand I, so the nucleation activity would be largely suppressed. However, the incumbent strand can still be exchanged. Victor I. Lyamichev et al. demonstrated that the overall replacement rate is a combination of two kinetic pathways: dissociative and sequential displacement (Figure 1b) [17]. In the dissociative pathway, the incumbent strand needs to be released from the duplex complex and thus exposes its unpaired bases ready for the nucleation process. As for the sequential displacement mechanism, the termini base pairs of original double-stranded complexes are easily frayed by thermal fluctuation. It is those metastable opened base pairs that create the foothold to promote the nucleation step. Once the invading strand binds, the incumbent strand is displaced by branch migration—a random walk with no net energy change and equal forward and backward probabilities [18,19]. The displacement process continues until the ends are met and the displaced strand detaches from the newly formed duplex. Qualitatively, the difference between these two pathways is the activation degree of the prehybridized complementary strand. Obviously, the dissociative mechanism requires higher energy input, and the other pathway is dominant under physiological conditions. According to the form of the invading strand, three-way and four-way (holiday junction, Figure 1c) branch migrations are the two general types [3]. The branch migration process is ubiquitous in living organisms as it plays a key role in DNA or RNA replication and genetic recombination [20,21,22]. This branch migration technology has been well-investigated and utilized decades ago [19,22], but the attention was mainly on its biological aspects.

Since the foundation of hybridization and strand displacement reactions is the formation of double-stranded complexes, factors affecting the stability of the duplex will perturb the reaction kinetics and yield as well. Typical factors including the sequence length, GC content, temperature, ionic strength, pH, molecular crowding reagents, solvents, water molecules, etc. Readers can find the excellent review by Wong et al. for more information [4].

### 2.2. Toehold-Mediated Strand Displacement Reaction

**Figure 2:**
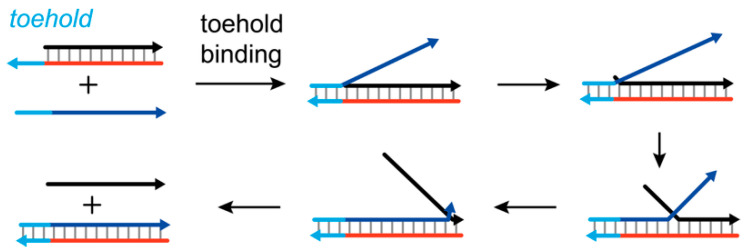
Toehold-mediated strand displacement (TMSD).

For SDR, producing well-defined duplex structures is at the cost of slow kinetics or rigorous environmental settings. Researchers have sought mechanisms that allow nucleation to occur at defined sites and reliably trigger branch migration. Until the year 2000, Yurke et al. employed branch migration reaction to build artificial nanomachines by DNA strands only. In their design, the target strand is extended longer than its incumbent counterpart, and the exposed single-stranded form tail is ready for nucleation with an invading strand. The underlying principle is analogous to introducing artificially extended “fraying tails” into sequential displacement mechanisms. By doing so, the strand displacement reaction is effectively activated. Since then, a new field, dynamic DNA nanotechnology, was opened and the basic branch migration reaction was given a later famous name, toehold-mediated strand displacement (Figure 2) [23].

As a building block to construct well-defined dynamic processes or nanomachines, the thermodynamic and kinetic properties of TMSD have been investigated till today. Several reviews have already summarized the design principles and applications in areas such as biosensing and nanostructure construction [2,3,5]. Briefly, the TMSD process can be modeled as a three-step reaction [14]. Firstly, the invader binds to the toehold region in the target strand, and finishes the toehold hybridization step rapidly due to short toehold length. Then, the branch migration proceeds as general strand displacement does. In the end, the incumbent strand detaches from the junction complex. Usually, the rate limiting process is the toehold hybridization step, which makes the overall displacement reaction fit second-order fashion. The simple model is represented as Equation (1).

(1) $$\begin{array}{c} I+\mathrm{TC}\overset{k_{\mathrm{TMSD}}}{\to }\mathrm{IT}+C \end{array}$$

Owing to the gaining of base pairs in the product duplex compared to its prehybridized TC reactant, the free energy change of the TMSD reaction is negative. Therefore, the typical TMSD reaction is regarded as irreversible. To quantitatively characterize the thermodynamic properties, a nearest-neighbor model has been developed and well-established [24,25,26,27,28,29]. Calculation software such as NUPACK has already shown excellent predictive capability on minimum free energy structures and melting temperatures [30,31]. With the increasing complexity of DNA nanotechnologies, determining the thermodynamic parameters of non-canonical base pairs and chemical modifications becomes demanding. David and Li groups proposed using DNA dynamic reactions as tools to measure the thermodynamic quantities [32,33].

In contrast, studying TMSD kinetics is more challenging. Researchers often use Markov-chain stochastic models or coarse-grained molecular dynamics to simulate the process [34,35]. Fluorophore-labeled oligonucleotides and the Förster resonance energy transfer (FRET) mechanism are usually used as an experimental validation method in bulk solution. Single molecule techniques such as atomic force microscopy allow for direct observation of TMSD isolated from bulk inferences [36]. Based on abovementioned algorithms, calculation packages such as Multistrand and oxDNA have been developed. To circumvent the algorithm challenges caused by different oligonucleotide sequences, secondary structures, and environmental factors, Zhang et al. proposed a weighted neighbor voting (WNV) prediction algorithm, a machine learning-wise strategy to predict kinetic rates from already-known rate values of similar reactions [37]. Recently, non-canonical TMSD reactions in the presence of base pair mismatches, heteroduplexes of RNA:DNA or PNA:DNA, and out-of-equilibrium RNA mixtures have been investigated [38,39,40,41]. Despite its critical importance, the impact of random sequence crosstalk within complex TMSD reaction networks remains underexplored. The Simmel group addressed this gap [42]. They systematically examined the kinetics of undesired reactions and proposed methods to increase the robustness of TMSD against molecular noise.

## 3. Activation and Regulation Strategies

Strand displacement can also be regulated by additional principles, such as molecular interactions or enzymatic assistance. Not limited to intrinsic nucleic acid base-pairing, molecular interactions, non-canonical association strategies, and auxiliary enzymatic methods will be discussed. A comparative summary of the different strategies, including their mechanisms, characteristics, and applications, is provided in Table S1 (Supporting Information). In addition, physical stimuli-responsive approaches such as light, thermal, magnetic, electrostatic, and ultrasonic cues can achieve strand displacement as well, but are not highlighted here [43].

### 3.1. Canonical Toehold

#### 3.1.1. Toehold Length

**Figure 3:**
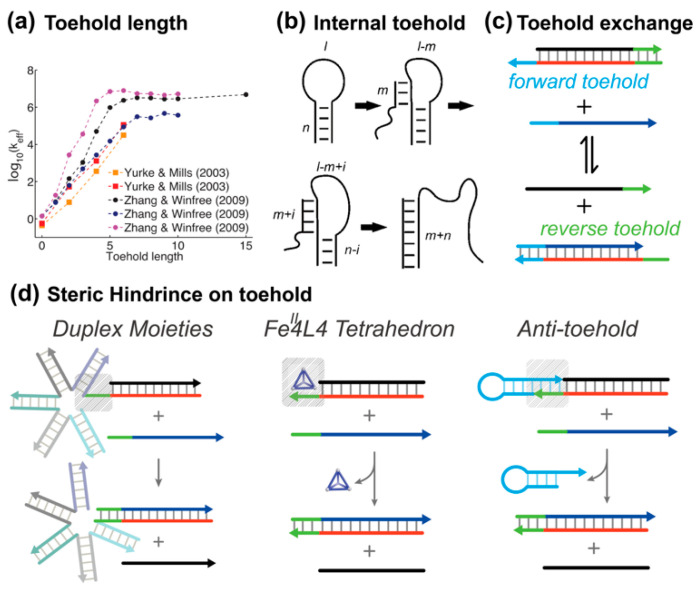
Tuning the activity of toehold via base-pairing and molecular interactions. (**a**) The displacement rate changes over six orders of magnitude by tuning the length of a toehold. Reprinted with permission from ref [34]. Copyright 2013, Oxford University Press. (**b**) Schematic depiction of an internal toehold. Adapted with permission from ref [44]. Copyright 2016, Biophysical Society. (**c**) Toehold exchange reaction. (**d**) Illustration of regulating the activity of a toehold via structural hindrance.

The simplest and most straightforward method for tuning the feasibility of creating proximity is changing the toehold length. Short toeholds can support nucleation, but their weak stability increases the chance of strand dissociation. The toehold must be long enough for branch migration to begin more quickly than the toehold can detach. Zhang et al. quantitatively studied the effect of toehold length on the kinetics of strand displacement rate [14]. Through the three-step simulation to fit the TMSD reaction, they found that the effective displacement rate is proportional to the length of the toehold in a logarithmic form (Figure 3a). When the toehold is 6 nt long, the best-fit rate constant is $1.0\times 10^{6}\;M^{-1}s^{-1}$, and the rate constant becomes saturated if the toehold binding energy exceeds −10 kcal/mol.

#### 3.1.2. Internal Toehold

The position of the toehold also influences the kinetics of strand displacement reactions. Green et al. demonstrated that the rate of loop opening is significantly faster when an external toehold is used compared to an internal one, with the reaction occurring 10–100-fold more rapidly in the case of external toeholds (Figure 3b) [44]. This is primarily due to the reduced entropic barrier when the toehold is external, as it avoids the topological constraints present when the toehold is internal. In contrast, an internal toehold constrains branch migration through the neck domain, which slows the reaction, particularly when the loop is long. This distinction in reaction kinetics is clear in experiments, where the displacement reaction initiated by an external toehold is nearly independent of the loop length within a certain range, while the reaction rate for internal toeholds significantly decreases as the loop length increases.

#### 3.1.3. Toehold Exchange

In a typical TMSD the direction of reaction is almost irreversible due to the overall free energy gain. To activate the reverse action, Zhang et al. proposed the toehold exchange design, where a second reverse toehold domain was embedded into the prehybridized duplex [14]. As shown in Figure 3c, the forward reaction is initiated by the collision of the forward toehold, and the reverse toehold remains inactive in the meantime. Once the branch migration process is completed, the forward toehold becomes sequestered in the product, while the reverse toehold is exposed, enabling a potential backward reaction. Owing to the two individual toeholds design, the thermodynamic properties of the overall reaction can be easily tuned by adjusting the length and GC contents of each toehold, whereas the rapid kinetic features can still be maintained. That is, the thermodynamic and kinetic properties of this type of TMSD reaction are separated, which gives more freedom for researchers to design fast dynamic circuits.

#### 3.1.4. Steric Hindrance on Toehold

Based on the collision mechanism of second-order reactions, steric hindrance near the toehold can slow TMSD kinetics. This can be achieved by bulky substituents or by reversible blocking modules. Steric hindrance limits access to the toehold, slowing the binding of the invading strand. For instance, Graugnard et al. introduced a large structural moiety around the toehold region (Figure 3d). Through systematic investigation, they showed that the steric factors yielded up to a three orders of magnitude decrease in the reaction rate constant [45]. By utilizing non-covalent interactions between a water-soluble tetrahedral cage FeII_4_L_4_ and unpaired bases in nucleic acids, Keyser et al. successfully controlled the strand displacement kinetics and applied this mechanism onto mismatch detection [46].

Another example is anti-toehold (At), a plug-and-play module developed by the Li group, for reversible and continuous control of DNA strand displacement kinetics [47]. The structure of anti-toehold is a hairpin where a toehold-complementary overhang was extended from its stem. The anti-toehold module can metastably bind to the toehold domain in the complementary strand, and thus suppresses the probability of the nucleation step for invaders. Having formed the three-stranded complex, the anti-toehold hairpin is then stabilized by both the base-pairing and base-stacking interactions at two junctions. By doing so, the kinetics of TMSD can be continuous and fine-tuned without the efforts of reprogramming the sequences of TMSD systems.

### 3.2. Non-Canonical Toehold Interactions

Aside from nucleic acid base-pairing, non-covalent molecular interactions such as non-canonical nucleic acid motifs, metal–base coordination, guest–host interactions, and aptamer–target binding are serving as versatile tools to translocate DNA strands into proximity. These toolkits also broaden the application scope of the strand displacement reaction to non-nucleic acids substrates.

#### 3.2.1. Metallo Activation

**Figure 4:**
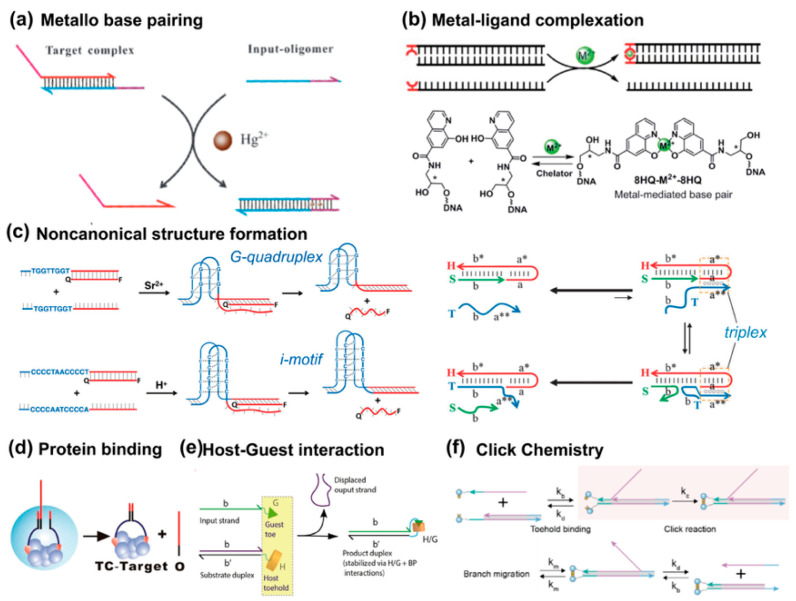
Non-canonical toehold interactions. (**a**) Metallo-base-pairing driven by Hg(II) ions. Reprinted with permission from ref [48]. Copyright 2013, Royal Society of Chemistry. (**b**) Metal–ligand complexation. Reprinted with permission from ref [49]. Copyright 2021, Royal Society of Chemistry. (**c**) G-quadruplex, and i-motif toeholds [50], reprinted with permission from ref [51], copyright 2013, American Chemical Society; and triplex toehold reprinted with permission from ref [52], copyright 2014, Royal Society of Chemistry. The asterisk (*) denotes complementary sequences. (**d**) Protein binding toehold, reprinted with permission from ref [53], copyright 2013, American Chemical Society. (**e**) Host–guest interaction. Reprinted with permission from ref [54], copyright 2022, American Chemical Society. (**f**) Click toehold from ref [55], copyright 2025, American Chemical Society.

Metal cations capable of interacting with oligonucleotides have also been employed to construct toehold switch modules. For instance, Ding et al. proposed a metallo-toehold format, in which Hg^2+^ cations trigger proximity activation by forming thymine–Hg^2+^–thymine metallo-base pairs (Figure 4a) [48]. In this system, the toehold sequence remains inactive due to T:T mismatches. However, the addition of Hg^2+^ in solution activates the TMSD process. Furthermore, metal-mediated artificial base-pairing can be independently utilized by placing a ligand-type nucleoside as an independent toehold (Figure 4b) [49]. Rather than integrating the metal-mediated base pair directly into the DNA structure, this approach uses metal–ligand complexation as a separate entity to regulate strand displacement dynamics. For example, Cu^2+^ ions can coordinate with 8-hydroxyquinoline ligands to form a stable and reversible metal-mediated base pair, initiating strand displacement. This strategy provides a flexible and tunable system. The displacement reaction is governed by the coordination properties of the metal–ligand complex, which can be modulated by metal species, concentration, and pH. Metal-driven activation enables reversible control of strand displacement, broadening the design space of DNA nanotechnology.

#### 3.2.2. Non-Canonical Nucleic Acid Motifs

Non-canonical nucleic acid structures can be formed under specific oligonucleotide sequences or environmental conditions, where the Hoogsteen pairing plays a key role (Figure 4c) [50,51,52]. Instead of carrying the genetic information, those higher-ordered motifs are believed to express regulation functions. Therefore, they are ideal materials for constructing molecular switches responsive to specific stimuli such as pH and cations. G-quadruplex (G4) and i-motif structures are typical non-canonical DNA motifs formed by guanine-rich and cytosine-rich sequences, respectively. The G4 structure requires cations to stabilize its G-tetrad layers, and the i-motif needs to be in acidic condition. Therefore, they have always been used to build DNA-based switches to activate the toehold recognition. A representative work by Tang et al. demonstrated an activation strategy where the toehold binding step is not achieved through base-pairing but by the formation of DNA tetraplex structures [51]. A triplex refers to the specific structure by binding a third nucleic acid strand to a conventional B-form DNA duplex. Liu and Mao demonstrated that transient C-containing triplexes formed under acidic conditions can trigger irreversible strand displacement, enabling the detection of otherwise fleeting molecular events [52].

#### 3.2.3. Binding-Induced Proximity

The binding-induced DNA strand displacement strategy relies on the proximity created by another molecule binding to trigger DNA strand displacement reactions (Figure 4d) [53]. In a protein binding approach, two DNA motifs (OT and C) are designed to bind to the same target molecule through affinity ligands (e.g., biotin–streptavidin interactions). When the target molecule is present, it brings the motifs into close proximity, significantly accelerating the strand displacement between OT and C. This displacement releases the output DNA (O), which can then initiate further DNA assemblies or be used in signal output systems. This strategy offers the advantage of using protein binding to control DNA displacement, providing a highly specific and efficient mechanism that can be applied to construct dynamic DNA circuits or detection systems. Besides the protein binding, other molecules such as small molecule ligands can achieve the activation of a toehold.

#### 3.2.4. Small Molecule Interactions

Another paradigm of non-covalent interaction to activate proximity is the host–guest interaction. Kankanamalage et al. proposed a synthetic surrogate to displace the base pair format of toeholds, i.e., host–guest interaction between a supramolecular host cucurbit uril (CB [7])–guest interaction (Figure 4e) [54]. This strategy allows for precise control over the displacement process, as demonstrated by the modulation of strand displacement kinetics through changes in the input structure, guest molecules, or linker length. Since this strategy is independent of base-pairing, the reaction can be controlled by adjusting the input structure or solution conditions. Latterly, Zhang et al. further introduced a click chemistry-accelerated toehold exchange (CCATE) approach, combining TMSD with strain-promoted azide-alkyne cycloaddition (SPAAC) (Figure 4f) [55]. This integration accelerates strand displacement by about 1000-fold compared to conventional methods. It also stabilizes toehold binding and significantly enhances overall reaction efficiency. These advancements broaden the potential applications of DNA nanotechnology, allowing for faster, more reliable DNA logic gates, amplification circuits, and biosensing systems.

### 3.3. Remote Toehold

**Figure 5:**
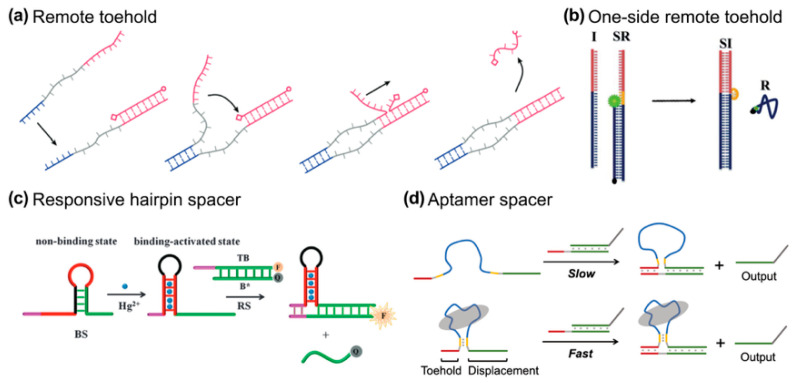
(**a**) Remote toehold, reprinted with permission from ref [56], copyright 2011, American Chemical Society. (**b**) One-side remote design, Royal Society of Chemistry. (**c**) Responsive hairpin as a spacer, reprinted with permission from ref [58], copyright 2015, Royal Society of Chemistry. (**d**) Aptamer spacer [59], reprinted with permission from ref [60], copyright 2021, Springer.

Since the toehold and branch migration domains are covalently hinged in a TMSD format, the invader and its complementary duplex are dragged into proximity once the toehold binding procedure is complete. To separate the toehold binding and branch migration processes, spacer regions can be inserted into the sequences of invader and complementary strands (Figure 5a). This particular design is termed remote toehold by Genot et al. [56]. They demonstrated that modulation of the nature and length of the spacer can control strand displacement rates over at least three orders of magnitude. Apart from the dual-side spacing design, Li et al. proposed a “one-sided remote toehold” strategy, where the spacer insertion is on the complementary strand [57]. After the TMSD process, a bubble secondary structure will be produced (Figure 5b). In this design, the toehold region becomes more sensitive to thermodynamic changes than normal TMSD does. This might be because of the increasing spatial freedom for the detached toehold domain.

External stimuli, such as metal ions or aptamer binding, have been shown to modulate remote toehold designs, offering tunable control over reaction kinetics. In particular, the use of conformational changes driven by external ligands or environmental conditions (e.g., small molecules or metal ions) provides a sophisticated means of adjusting the toehold spacing and, consequently, the kinetics of strand displacement. Zhu et al. introduced a metal ion or ATP-triggered hairpin reconfiguration to modulate the distance between the toehold and branch migration domains, offering a tunable reaction rate that can be fine-tuned via external stimuli (Figure 5c) [58].

Aptamers are single-stranded nucleic acids that fold into specific structures and bind targets with high affinity and specificity. Zhang et al. extended this concept by embedding an aptamer sequence between the toehold and displacement domains [59]. Therefore, ligand binding brings the domains together and accelerates the strand displacement reaction. This approach shows how ligand-induced proximity can enable efficient and controlled DNA circuits. Similarly, Lai et al. employed a conformational motion strategy (InCMS), where a reconfigurable hairpin loop is used to adjust the spacing, allowing for a broader kinetic range by changing the spacer length (Figure 5d) [60]. Unlike static spacers, these dynamic systems open new avenues for precision control in DNA-based devices, where the spacer length and conformation can be actively manipulated, offering both flexibility and responsiveness.

### 3.4. Associative Activation

**Figure 6:**
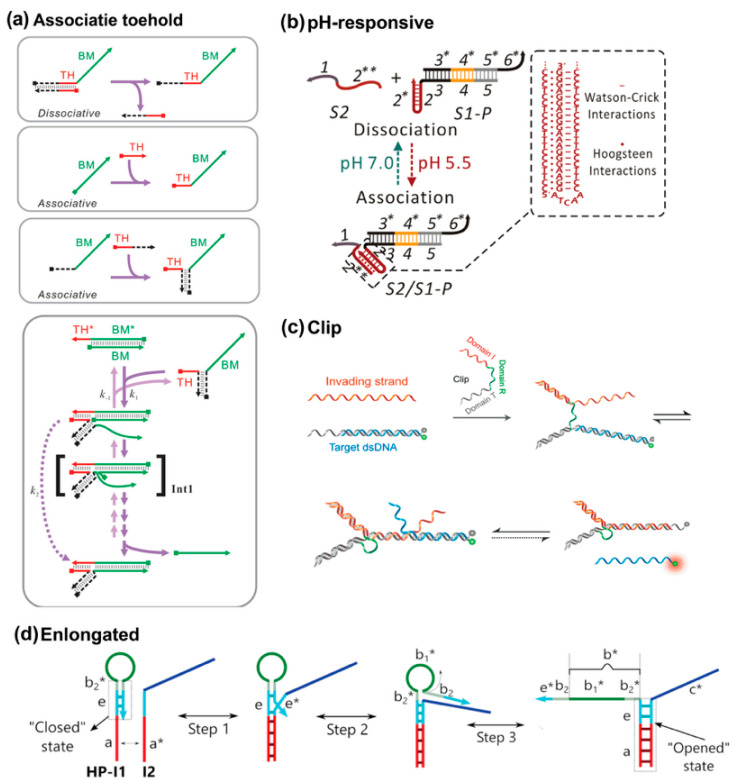
(**a**) Schematic illustrations of associative activation [61,62,63,64,65,66,67,68,69]. Reproduced with permission from ref [61], copyright 2011, American Chemical Society. (**b**) pH-responsive association. Reproduced with permission from ref [64], copyright 2020, American Chemical Society. (**c**) Clip associative strategy, reproduced with permission from ref [63], copyright 2021, American Chemical Society. (**d**) Elongated associative toehold, reproduced with permission from ref [69], copyright 2021, Oxford University Press.

For the standard TMSD reaction format, the toehold and branch migration regions are covalently linked. Even for the remote toehold design, the spacing remains with a resealable distance. However, this hard-wired linkage restricts the design space because the sequence of strands is definitive after chemical synthesis. To expand the design space, Xi Chen developed the associative principle, in which the toehold and branch migration domains are completely divided as two individual species (Figure 6a) [61]. The invader is unable to displace the incumbent strand, yet binding with toehold species only has nothing to do with displacement reactions either. This shows that proximity drives strand displacement, while toehold hybridization is just one possible mechanism. Therefore, an auxiliary complementary domain is added to the toehold and branch migration species, such that the toehold can be assembled with the BM species via hybridization. Xi Chen’s work represents a conceptual shift by decoupling recognition from branch migration. This design allows unprecedented modularity and offers the possibility of more dynamic regulation of reaction kinetics.

Various strategies for further expansion have been developed. One of them is introducing alternative associative interaction through the auxiliary domain, including immunobinding events [62,63], aptamer–ligand interaction [60], pH-responsive triplex formation [64,65], and cooperative base-stacking [66]. For example, in Figure 6b, triplex design allowed the reaction to be toggled on and off depending on the pH, offering a simple and effective way to regulate strand displacement while avoiding the production of additional waste DNA strands [65]. More complicated associative strategies have also been investigated. Xiao’s lab developed a wedge-like DNA tool, which allows for bidirectional regulation of the displacement reaction, offering precise control over the reaction rate by adjusting the length and orientation of the wedge domain [67]. They further introduced the Clip tool, which not only regulates the displacement rate through a similar modular approach but also offers additional functionalities such as allosteric activation, selective pathway control, and resettability (Figure 6c) [68]. The Clip design operates independently of the invading and template strands, ensuring high compatibility with existing DNA devices while expanding the range of possible applications in regulating the strand displacement reaction. Further advancing this concept, Ang and Yung developed a dynamically elongated associative toehold that utilizes a hairpin lock to control the length of the association domain in response to input targets (Figure 6d) [69]. This design reduced leakage in the circuit by keeping the association region short in its initial state and elongating it upon target binding, thereby stabilizing the strand displacement reaction. The dynamic elongation not only improved the reaction kinetics but also enhanced the equilibrium signal, making this strategy a powerful tool for optimizing DNA circuits that require precise control over both the kinetics and thermodynamics of the reaction.

### 3.5. Hierarchical Activation

**Figure 7:**
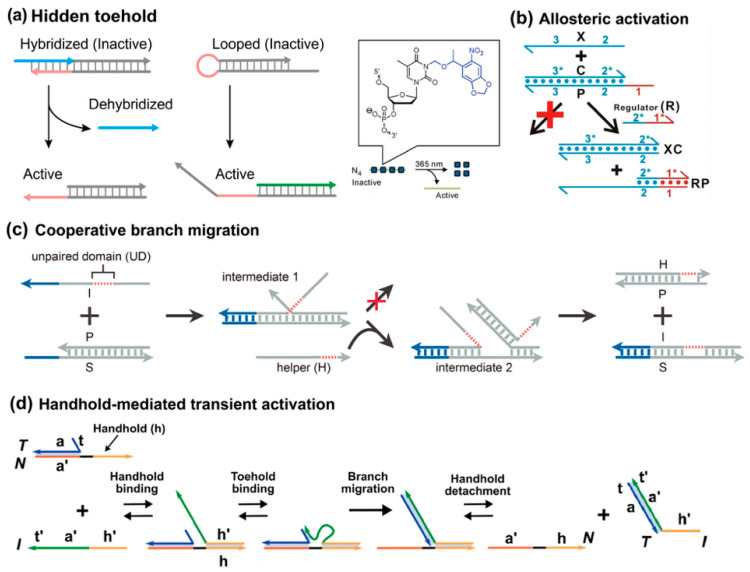
Hierarchical activation. (**a**) Toeholds were hidden by hybridization, as well as loop and photocaged modification, reproduced with permission from ref [71], copyright 2015, American Chemical Society. (**b**) Allosteric toehold activation, reprinted with permission from ref [72], copyright 2016, American Chemical Society. (**c**) Cooperative branch migration, reprinted with permission from ref [73], copyright 2022, American Chemical Society. (**d**) Handhold-mediated strand displacement, reprinted with permission from ref [74], copyright 2021, American Chemical Society.

Previous strategies are mainly dependent on single-step collision or assembly. In hierarchical designs, the whole strand displacement process is prolonged with an intermediate activation step. A typical format of this principle is hiding the toehold at first and then activating it by activators. In this design, the toehold can be initially hidden by other blockers, such as hybridization, as well as hairpin and photocage modification (Figure 7a) [70,71]. A flexible format of exposing the hidden toehold is allosteric toehold, proposed by the Li group in 2016 (Figure 7b) [72]. The single-stranded invader does not possess any sequence foothold with its complement, yet the prehybridized duplex still has an overhang domain. In this case, the strand displacement reaction cannot occur effectively without an external regulator. At first, the regulator attaches to the overhang sequence and then undergoes a typical branch migration process. However, the regulator is unable to displace the incumbent strand, thus forming a three-stranded complex intermediate. As such, a toehold domain can be created for the invader within this intermediate, and only then the typical TMSD mechanism can occur. A characteristic difference in this allosteric toehold is that the overhang domain is extended from the incumbent strand, rather than the complementary strand. One defect of allosteric toehold is the metastability of the intermediate; as such, an extremely high ionic strength condition is required to prolong its lifetime. Lai et al. utilized secondary structure to stabilize the intermediate [59].

Besides the hierarchical activation of toeholds, the Xie group proposed another mechanism to achieve hierarchical activation of branch migration, termed cooperative branch migration (Figure 7c) [73]. In this design, the invader cannot reach close proximity with its complementary duplex and cannot successfully complete the branch migration process due to an intramolecular spacer. To help overcome the structural barrier, a helper strand participates to hybridize with the pried incumbent strand in an intermediate complex.

A recent advancement in hierarchical activation is the concept of handhold-mediated strand displacement (HMSD), introduced by Cabello-Garcia et al. in 2021, which enhances the versatility of DNA strand displacement reactions by introducing a transient recognition interaction with an independent overhang, or handhold, in the incumbent strand (Figure 7d) [74,75]. Unlike traditional TMSD, where the toehold remains cooperatively sequestered in the product, HMSD allows for reversible binding of the invader to the handhold, which accelerates the displacement reaction without being incorporated into the final product. Because the product detaches from the template, the system operates far from equilibrium, resembling biological templating processes. This system has been shown to enhance the displacement rate by up to four orders of magnitude, especially for systems with short toeholds, where handholds significantly increase the effective concentration of the invader strand in the vicinity of the target. Moreover, HMSD facilitates the templating of specific DNA duplexes, generating a nonequilibrium product ensemble that mirrors processes seen in biological templating mechanisms like RNA transcription, where transient interactions lead to highly specific, out-of-equilibrium products. This enables more versatile designs for creating complex DNA structures and circuits that operate in dynamic environments, where templates can be reused, and products can be generated with increased specificity and efficiency. It shows transient proximity can accelerate displacement kinetics, while maintaining thermodynamic neutrality.

Overall, hierarchical activation strategies regulate strand displacement by introducing intermediate steps. Designs based on hidden or allosteric toeholds give modular control but can be hampered by the instability of transient intermediates. Cooperative branch migration relies on helper strands to overcome structural barriers, while handhold-mediated displacement greatly accelerates reactions and enables template reuse, though it requires more careful design. These different routes illustrate how multi-step mechanisms can tune both the timing and the efficiency of SDR.

### 3.6. Toehold-Free Strand Displacement

Toehold-free strand displacement (TFSD) represents an emerging and innovative approach in the realm of DNA nanotechnology, offering distinct advantages in terms of both flexibility and functionality compared to traditional toehold-dependent systems. In conventional TMSD, the interaction is initiated through a short single-stranded overhang, or toehold, that facilitates the invasion of one strand into a duplex. The toehold reduces the energy barrier and improves specificity, but in complex systems it may also slow reactions and increase nonspecific binding. The concept of toehold-free strand displacement circumvents these challenges by leveraging alternative mechanisms to drive DNA strand exchange. Herein we introduce the non-enzymatic toehold-free solutions to achieve strand displacement purposes.

**Figure 8:**
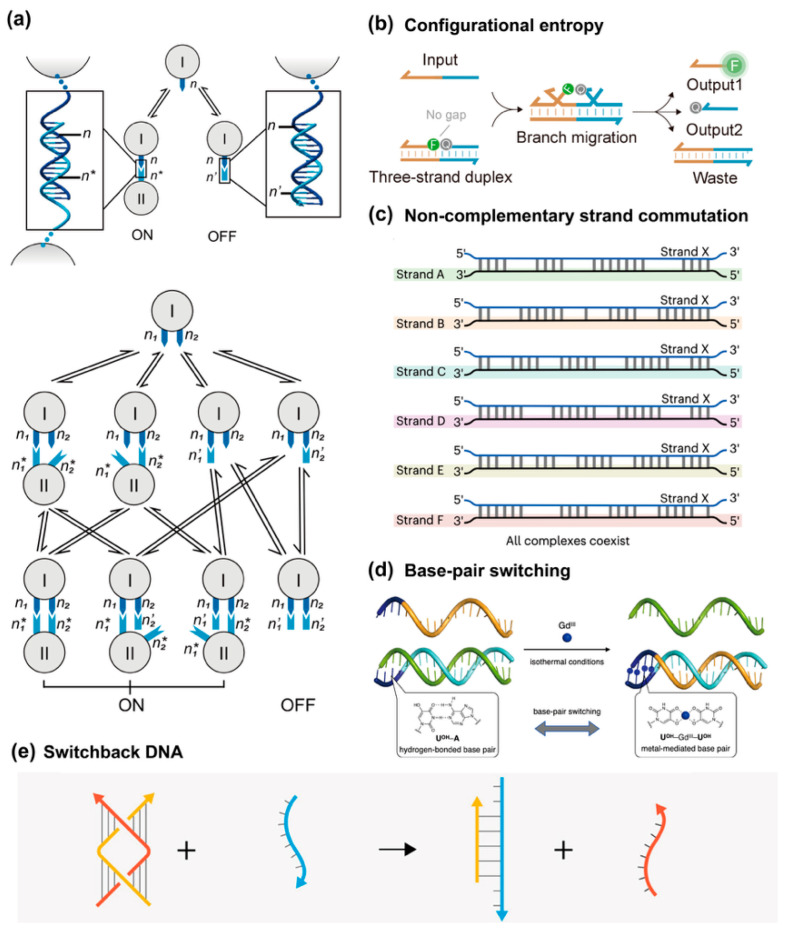
Enabling strand displacement reaction through non-enzymatic toehold-free approaches. (**a**) SDRs are driven by multivalent interaction and concentration imbalance, reprinted with permission from ref [76], copyright 2021, Springer. (**b**) Configuration-entropy-driven SDRs, reprinted with permission from ref [77], copyright 2025, American Chemical Society. (**c**) Non-complementary strand commutation, reprinted with permission from ref [78], copyright 2021, Springer. (**d**) Reprinted with permission from ref [79], copyright 2023, Springer. (**e**) Switchback DNA strand displacement, with permission from ref [80], copyright 2025, American Chemical Society.

One solution is utilizing the concept of multivalency by adhering strands within a confined space such that the diffusion barrier in bulk solution can be largely eliminated. The Wei group demonstrated this idea in the year 2021 (Figure 8a) [76]. With the help of well-constructed DNA nanostructures, two complementary strands (immobilized n_1_ and n_1_*) are inserted onto two origami units. Under the condition of excess of single-stranded species (free n_1_*) in bulk solution, those two origami units cannot interact with each other. To initiate multivalent strand displacement, another set of complementary strands (immobilized n_2_ and n_2_*) should be decorated at the surface of origami units as well. Hybridization of immobilized n_2_ and n_2_* drags the distance between two origami units and closes the proximity between an immobilized n_1_n_1_* duplex and immobilized n_1_*. Even though no toehold sequence exists for strands n_1_ and n_1_*, the strand displacement reaction can occur through fraying ends. Also, this work showed the driving force of concentration imbalance.

The second primary force behind TFSD is entropic gain, rather than the enthalpic reduction associated with toehold hybridization. The TFSD can be driven by configurational entropy of single-stranded DNA during the displacement process, which compensates for the absence of a toehold (Figure 8b) [77]. This design not only removes the need for a specific overhang sequence but also opens up the possibility of using multiple strands to achieve the desired strand displacement, thus broadening the scope of DNA nanotechnology applications.

Another solution is to promote the sequential displacement pathway for mismatched complementary strands. The discovery history of toeholds and TMSD originated from the nature-inspired biological processes, where DNA strands were preferably to be in complementary form. But “the question arises of whether any other data are held by DNA in a non-traditional form—that is, unreadable within the current paradigm of the double helix, complementarity and the genetic code? If yes, then what would be the underlying mechanisms?” To answer the question, Maxim P. Nikitin systematically studied the reaction paradigm where strand displacement occurred between largely mismatched strands, termed strand commutation. Surprisingly, this seemingly simple and naïve reaction paradigm exhibits amazing potential in constructing fast and versatile DNA computing modules (Figure 8c) [78].

Base pair switching, particularly using metal ions as triggers, represents another approach in TFSD. Takezawa et al. introduced a metal-mediated base pair switching system that utilizes 5-hydroxyuracil (UOH) as a metal-responsive nucleobase (Figure 8d) [79]. In the presence of metal ions like Gd^3+^, UOH forms a stable metal-mediated base pair that can switch between hydrogen-bonded UOH-A and metal-bound UOH-GdIII-UOH pairs, driving the strand displacement process. This metal-induced switching mechanism not only allows for precise control over DNA displacement reactions but also expands the range of external stimuli that can modulate DNA nanodevices, making them more adaptable for real-world applications. Madhanagopal and Chandrasekaran also explored toehold-less strand displacement using a novel DNA nanostructure called Switchback DNA (Figure 8e) [80]. This structure is based on two DNA strands pairing in an unconventional manner that does not rely on traditional toehold-mediated displacement. In their study, they showed how altering the affinity of the Switchback DNA components allows for the reversible reconfiguration of the DNA nanostructure, using a third DNA strand that interacts with the structure but without requiring a toehold.

Toehold-free mechanisms broaden SDR by eliminating the need for predefined overhangs and instead exploiting proximity, entropy, or alternative base-pairing modes. Multivalent and entropy-driven designs offer flexibility but demand precise structural organization. Mismatch-based and metal-mediated strategies increase chemical diversity, though often at the cost of predictability and greater risk of cross-talk. Taken together, these approaches show the opportunities and constraints of building SDR systems without toeholds.

### 3.7. Enzyme Participation Methods

Owing to the biocompatibility of DNA/RNA oligonucleotides, enzymes are naturally suitable to manipulate the strand displacement process with synergistic functions that natural nucleic acid chemistry finds difficult to deal with.

**Figure 9:**
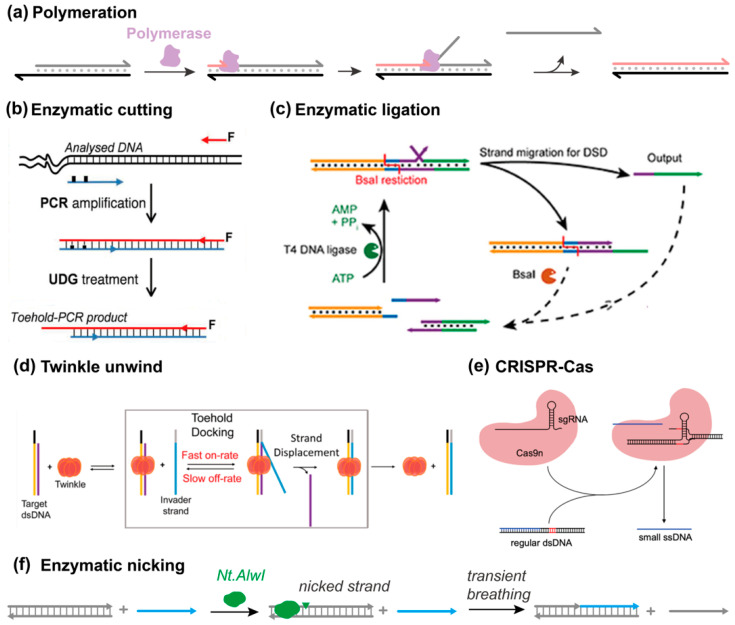
Enzymatic activation and regulation. (**a**) Achieving strand displacement via the polymerization approach from ref [81]. (**b**) Enzymatic cutting of toehold blockers, reprinted with permission from ref [83], copyright 2013, American Chemical Society. (**c**) Enzymatic ligation, reprinted with permission from ref [84], copyright 2020, American Chemical Society. (**d**) Twinkle-catalyzed SDRs, reprinted with permission from ref [85], copyright 2023, American Chemical Society. (**e**) CRISPR-mediated SDRs, reprinted with permission from ref [86], copyright 2021, American Chemical Society. (**f**) Nicking endonuclease-induced transient breathing, which activates the SDR, reprinted with permission from ref [87], copyright 2024, Springer.

Polymerase exhibits its capability to directly activate strand displacement. Usually, the strand displacement reaction is achieved via the branch migration process; that is, the sequence of the invader is pre-synthesized. On the other hand, strand exchange can be achieved on the fly along with the polymerization process (Figure 9a) [81]. In this case, the invader does not possess the oligonucleotide sequence identical with its incumbent counterpart but only the toehold domain. Like the primer working in a polymerase chain reaction, the invader rapidly hybridizes with the complementary strand and then the polymerase extends the invader along with the synthesizing process. In a typical TMSD reaction, the nucleation step of toehold binding is very sensitive to small thermodynamic changes, and this feature endows toehold exchange reaction capability in biosensing applications. Only “exact” toehold recognition can effectively trigger a displacement reaction. However, the polymerization mechanism is relatively “fuzzy” such that similar yet different inputs can accomplish an identical strand displacement process [82].

Several other enzymes are intended to activate the toehold hybridization step. For example, as early as the year 2013, Khodakov and Ellis et al. used uracil-DNA glycosylase (UDG) to create a toehold domain from double-stranded PCR amplicons (Figure 9b) [83]. By substituting deoxythymidine for deoxyuracil (dT→dU), the lesion after UDG treatment drove the oligonucleotide detachment. Similar actions can be achieved by nicking enzymes as well. In addition, enzymatic degradation is also an effective method to remove toehold blockers, which further leads to the dissipative DNA nanotechnology presented in Section 4 [84]. Except for cutting the toehold sequence out, ligase can be utilized to combine a separated toehold region with a branch migration substrate, resembling the associative mechanism, but at the cost of ATP hydrolysis (Figure 9c) [84]. Inspired by the natural replication process, Patel et al. harnessed the helicase Twinkle to unwind the duplex region from the single-stranded 5′-toehold end, as such greatly increasing the kinetics of TMSD (Figure 9d) [85]. With increasing attention being attracted onto CRISPR-Cas technologies, the potential of achieving strand displacement through CRISPR has been explored by Rodrigo et al. (Figure 9e) [86]. A recent work by Wei et al. demonstrated that toehold-free strand displacement can be achieved by exploiting transient base pair breathing at the ends of a DNA duplex (Figure 9f) [87]. DNA-modifying enzymes, such as nicking endonucleases, can enhance local breathing and promote strand exchange without the need for a conventional toehold. This mechanism enables the invading strand to initiate displacement at blunt-ended duplexes by capitalizing on the naturally occurring fluctuations in base-pairing.

Enzymes add catalytic control to strand displacement in ways that pure DNA designs cannot. Polymerases and ligases can couple displacement with synthesis or joining events, while nicking enzymes and helicases accelerate initiation by generating or exposing toeholds. CRISPR systems extend the range of possible triggers. Such methods are fast and versatile, but their reliance on protein cofactors can be a drawback for fully synthetic or minimal systems.

### 3.8. Non-Specific Global Tuning Factors

Aside from specific activation methods such as toehold recognition or molecular interactions, environmental factors including pH, temperature, metal cations, solvents, viscosity, etc. can affect the strand displacement reaction as well [4]. Since nucleic acid oligonucleotides are negatively charged, positively charged polymers can be used to neutralize the electrostatic force and accelerate the hybridization process. The Maruyama group reported a series of comb-type copolymers that can greatly enhance the kinetics of the strand displacement reaction. The general format of a polymer structure is using poly(L-lysine) as a backbone and grafted with dextran chains (PLL-g-Dex) [88,89].

Small molecule DNA binders are a class of chemicals that can bind to DNA through major or minor groove binding, electrostatic interaction, or intercalation. By harnessing the stabilization property of binder molecules onto DNA structures, Xu et al. proposed a unique programming strategy termed binder-induced nucleic acid strand displacement (BIND) [90]. In a solution containing extremely low concentrations of metal cations, double-stranded nucleic acids are in a metastable state where the dissociative pathway is the major strand displacement pathway. But with addition of binder molecules, the helical structures can be largely stabilized such that a toehold-mediated displacement is then preferrable. By combining with fluorescent intercalator displacement (FID) assay, this unique BIND mechanism can achieve high-throughput screening of binder molecules with higher fidelity.

## 4. Advanced Dynamic Control

The strand displacement behaviors discussed in the preceding subsections generally attain an equilibrium state, regardless of the complexity of individual activation mechanisms or reaction networks. Recent advances in DNA nanotechnology have expanded the functionality of strand displacement reactions (SDRs), transitioning from static logic operations to highly dynamic and temporally regulated processes. A pivotal development in this evolution involves sophisticated dynamic control strategies, which enable autonomous, reversible, and temporally precise responses in SDR systems. This section highlights three representative approaches that collectively exemplify the state of the art in this field. Given the comprehensive review papers available, however, only a concise overview of these topics is provided.

### 4.1. Nucleic-Acid-Only Network

To achieve intricate and autonomous temporal control, nucleic acid-based network regulation is a powerful approach that allows the construction of systems with modular and reprogrammable behaviors. The ability to encode complex dynamics, such as feedback loops, oscillations, and signal cascades, using purely nucleic acid species has been demonstrated in several groundbreaking studies [91,92,93].

**Figure 10:**
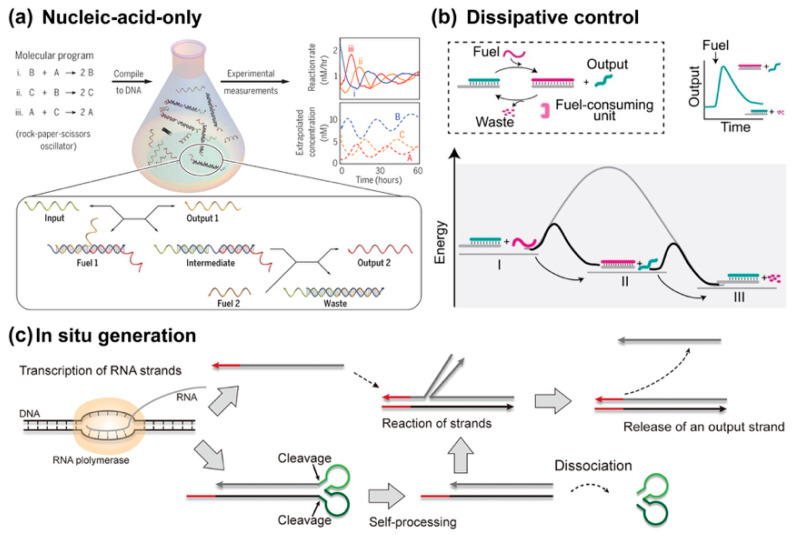
Advanced dynamic control. (**a**) Nucleic-acid-only dynamical system, reprinted with permission from ref [92], copyright 2017, AAAS. (**b**) Dissipative control by external stimuli, reprinted with permission from ref [94], copyright 2022, Wiley-VCH GmbH. (**c**) Producing components in situ, reprinted with permission from ref [95], copyright 2020, American Chemical Society.

A prominent example of such an approach is seen in the work by Srinivas et al., where a hierarchical molecular programming language was developed to create autonomous DNA oscillators (Figure 10a) [92]. By leveraging TMSD reactions and formal chemical reaction networks (CRNs), they successfully implemented a rock–paper–scissors oscillatory circuit using only DNA components. In this design, DNA strands are manipulated to mediate cascading reactions, where the concentration of species fluctuates over time according to a cyclic competition mechanism. This work not only demonstrated complex temporal dynamics in synthetic nucleic acid systems but also highlighted the scalability of the approach, enabling the design of more intricate systems that can exhibit even more sophisticated behavior, such as multi-input feedback loops and signal amplification. With the ability to programmatically encode these dynamics into the DNA sequences themselves, the regulation of such systems becomes highly controllable, creating potential for applications in diagnostics, therapy, and synthetic biology [3].

Despite significant progress, all-DNA dynamic systems remain constrained by intrinsic limitations. Most rely on strand displacement cascades that proceed irreversibly, yielding functions that are essentially single-use unless externally reset. Fuel strands act mainly as triggers rather than sustained energy sources, so the dynamics are transient, and energy utilization is inefficient, with much lost as byproducts. Waste accumulation, incomplete reactions, and fuel degradation further compromise reliability and prevent repeated operation. As a result, current devices generally toggle between only a few states and exhibit limited functional complexity. Advancing the field will require strategies to improve reversibility, enhance energy efficiency, reduce waste, and support more sophisticated behaviors for robust and practical applications [96].

### 4.2. Dissipative Control by External Stimuli

The emerging field of dissipative DNA reaction networks represents a groundbreaking frontier in dynamic DNA nanotechnology, aiming to emulate transient biological processes that operate out-of-equilibrium through continuous energy consumption. These networks leverage the exceptional programmability of DNA molecules to create synthetic systems capable of exhibiting life-like behaviors such as adaptation, memory, and autonomous control [96,97,98,99,100,101]. Unlike equilibrium-driven DNA assemblies, dissipative networks require constant energy input to sustain transient states, enabling dynamic functions that closely mimic natural biological pathways. The fundamental significance of these systems lies in their capacity to bridge the gap between static nanostructures and adaptive molecular systems, offering profound insights into the principles of biological organization while providing innovative tools for creating responsive molecular devices.

It is crucial to emphasize that while a dissipative reaction network constitutes a dynamic system, not all dynamic circuits, even those exhibiting oscillatory or temporal behaviors, qualify as dissipative systems. Ricci provides a comprehensive and lucid explanation of this distinction [96]. The core concept involves converting the chemical energy stored in fuel species into waste while performing work during the process. Importantly, the entire system, apart from the fuel and waste, must return to its original state after the dissipation cycle. As for the pivotal energy conversion step, it can be achieved through various enzymatic means such as light [102,103,104,105], protein enzymes (e.g., polymerase, ligase, RNase H, lambda exonuclease) [106,107,108,109,110,111], or through DNAzymes [103,112,113].

The Ricci group proposed the most straightforward format of dissipative TMSD reaction (Figure 10b) [94,96]. The original state of the system contains a prehybridized complementary duplex and RNase H endoribonuclease (RNase H). Once the RNA fuel strand enters the system, it rapidly undergoes the TMSD reaction and forms the temporal product RNA:DNA heteroduplex. Then RNase H slowly degrades the RNA duplex species into waste. Given enough time, such that all RNA fuels are consumed to waste, this system returns to its original state, where the incumbent strand is rehybridized to its complementary strand. Since this mechanism is self-repetitive, this whole system is out-of-equilibrium at any time. The energy dispersed originates from the hydrolysis of fuel strand to waste. In the same year, they demonstrated another TMSD activation control via a dissipative blocking strategy [114]. In this design, an RNA blocker strand temporally inhibited the toehold region of TMSD; meanwhile, this very blocker is the fuel species that will be gradually consumed. As such, the TMSD can be temporally controlled by the quantity of fuel. Through cascading dissipative systems, they successfully constructed a programmable timed pulse signaling pattern [115].

Aside from the protein enzymes, DNAzymes are always used to construct dissipative networks as well [113,116]. DNAzymes are synthetic single-stranded DNA sequences that exhibit diverse catalytic functions including RNA cleavage, DNA ligation, and phosphorylation [103]. The Willner group went to great effort to construct DNAzyme-based dissipative circuits [98]. The basic format includes an inactive blocker–prehybridized DNAzyme duplex and a pair of short single-stranded signal reporters competing to bind with the blocker. Addition of the fuel simultaneously displaces the blocker strand and drives the formation of an active DNAzyme state. At this very state, the reporters bind with the blocker and the fuel is gradually depleted into wastes. Once the fuel is completely cleaved into two short waste strands, the entire system reverts to its original state [98,103]. As an example of demonstrating the application potential of the dissipative strand displacement mechanism, Wu et al. utilized the DNAzyme as the invader in a toehold-mediated strand displacement reaction and achieved a time-controlled logic gate [116]. Thereafter, they applied this framework to construct temporal Boolean logic authentication and access [117]. The Herrmann group successfully achieved controlled release of therapeutic nucleic acids. Cytosine–phosphate–guanine (CpG) oligodeoxynucleotides (ODNs) are a class of single-stranded DNA that exhibit high immunostimulatory characteristics [110,118]. Since the CpG ODNs should be protected against cleavage, the authors chemically modified the sequence with phosphorothioate, and bound and utilized a hairpin species as fuel. This work paves the way for the design of smart drug delivery systems based on dissipative strand displacements.

Dissipative DNA reaction networks provide a transformative approach for engineering dynamic molecular systems with life-like properties. Through strategies like enzyme- and light-driving, they achieve non-equilibrium behaviors for applications in biomedicine and smart materials. Overcoming challenges in scalability, robustness, and energy efficiency will be crucial to fully realizing their potential in creating adaptive, intelligent systems that bridge synthetic chemistry and biological complexity.

### 4.3. In Situ Generation of Nucleic Acid

A major challenge for nucleic acid circuits is the degradation of fuel strands and the difficulty of generating required components in situ. Recently, the generation of nucleic acid complexes in situ represents a powerful approach to dynamic control in strand displacement reactions, particularly in systems requiring autonomous, self-sustained functionality.

Bae et al. introduced a key innovation in this area by utilizing self-cleaving ribozymes to autonomously produce RNA-based gate complexes (Figure 10c) [95]. Their approach involves the transcription of a long RNA strand, which folds into a multistranded complex and cleaves itself at defined locations via ribozymes, creating the requisite components for strand displacement reactions. This method eliminates the need for pre-synthesized or externally introduced components, enabling the continuous operation of nucleic acid circuits within closed environments, such as living cells or artificial reaction chambers. Then, Schaffter and Strychalski developed cotranscriptionally encoded RNA strand displacement (ctRSD) circuits, where the components necessary for reaction are generated isothermally during transcription [119]. By embedding self-cleaving ribozymes within the circuit design, the RNA gates can fold cotranscriptionally, ensuring precise stoichiometry and preventing unwanted leaks typically associated with separate transcription steps. This ctRSD system allows for the autonomous assembly and operation of complex molecular circuits, capable of executing logic, amplification, and cascading reactions entirely within the transcription environment. Notably, the kinetics of these circuits are predictable through a simple model that couples transcription and strand displacement, providing a robust framework for the design and scaling of molecular computation in biological settings.

These studies illustrate progress toward SDR systems with temporal control, autonomous function, and programmable structures. They lay a foundation for designing adaptive molecular controllers with potential applications in synthetic biochemical regulation.

## 5. Conclusions

In this review, we have summarized proximity activation strategies for controlling strand displacement reactions (Table S1). By tracing the principles of nucleic acid hybridization, spontaneous pathways, and toehold-mediated displacement, we emphasized that spatial proximity provides a unifying perspective across different mechanisms. Building on this view, we classified SDR methods according to their nucleation principle: canonical toehold-based reactions represent proximity activation through base-pairing, while non-canonical strategies rely on alternative molecular interactions. This perspective emphasizes that toehold binding is only one of several ways to achieve displacement, and helps explain the emergence of “toehold-free” systems.

We also highlighted recent progress in dynamic control, particularly dissipative DNA nanotechnology, where energy-consuming circuits allow temporal regulation and autonomous operation. These advances point toward more dynamic, programmable, and application-ready SDR systems for biosensing, computation, and therapeutic delivery.

Finally, although not addressed in prior sections, predicting hybridization kinetics or TMSD rates directly from sequence remains a significant challenge. Early work, such as the mentioned WNV algorithm developed by Zhang and colleagues, used curated experimental datasets to estimate hybridization rates [37]. More recently, machine learning has been introduced to this field. Models including decision trees, random forests, convolutional networks, and transformer architectures have been trained on sequence-derived features such as toehold length, GC content, and hydrogen-bonding patterns to predict rate constants with higher accuracy [42,120,121,122,123]. Examples include decision-tree models for external toehold protection strategies [42], random forest analyses of rate-determining features [123], and neural network models that integrate molecular dynamics simulations to predict nuclease activity in CRISPR systems [124]. These studies illustrate the broader promise of computational tools in guiding the rational design of SDR circuits and functional nucleic acid devices.

Despite considerable advances in strand displacement technologies, several fundamental challenges must be resolved to enable their broad application. A primary obstacle involves ensuring robust operational stability under physiological conditions, which demands improvements in resistance to nuclease degradation, nonspecific binding, and dynamic environmental fluctuations. Additionally, achieving high fuel strand efficiency while minimizing the accumulation of inert or inhibitory byproducts remains critical for sustaining prolonged functionality. The development of predictive theoretical frameworks represents another urgent priority; such models must accurately correlate sequence design with reaction kinetics and thermodynamic behavior to support reliable system optimization.

In terms of near-term applicability, biosensing and diagnostic platforms currently exhibit the most immediate potential for real-world deployment, owing to their modularity and compatibility with existing readout technologies. In contrast, applications in therapeutic delivery and in vivo programming necessitate further breakthroughs in biological stability, targeted controllability, and intracellular compatibility before they can transition into practical use.

## Data Availability

Not applicable.

## Supplementary Materials

The following supporting information can be downloaded at: https://www.mdpi.com/article/10.3390/bios15100683/s1, Table S1: Comparison of activation and regulation strategies.
