# Supplementary material for: Kinetics and Activation Strategies in Toehold-Mediated and Toehold-Free DNA Strand Displacement
Source: Biosensors (Basel). 2025 Oct 9;15(10):683. doi: 10.3390/bios15100683 (PMC12562861; doi:10.3390/bios15100683)
Supplement: Supplementary file 1 [file biosensors-15-00683-s001.zip › biosensors-3879123-supplementary.pdf]

biosensors MDPI

Supporting Information

Kinetics and Activation Strategies in Toehold-Mediated and Toehold-Free DNA Strand Displacement

Yuqin Wu †, Mingguang Jin †, Cuizheng Peng †, Guan Alex Wang * and Feng Li *

Key Laboratory of Green Chemistry and Technology of Ministry of Education, College of Chemistry, Sichuan University, Chengdu 610064, China
* Correspondence: guanalexwang@scu.edu.en (G.A.W.); windtalker_1205@scu.edu.cn (F.L.)
† These authors contributed equally to this work.

Table S1. Comparison of activation and regulation strategies.

Category	Mechanism	Characteristics	Applications
Canonical (proximal) toehold	Hybridization via designed single-stranded DNA (ssDNA) overhangs; Tunability through length/GC content, position (external vs. internal), toehold exchange, or steric blocking	Fast activation; coarse tuning by length/GC; finer adjustment through position and steric effects; toehold binding is often the rate-limiting step; toehold exchange enables reversibility	Biosensing, DNA circuits, nanomachines
Non-canonical toehold interactions	Metal–base or metal–ligand coordination; G4/i-motif/triplex (Hoogsteen); aptamer–ligand; host–guest; click-assisted	Stimuli-responsive; fine tuning via stimuli/competitors; base-pair orthogonality (e.g., host–guest interactions); context-dependent	Responsive sensors, smart switches, controllable logic modules
Remote toehold	The toehold and branch migration (BM) domain remain linked but separated by a spacer (distance adjustable)	Kinetics are tunable across orders of magnitude by spacer length and structure; achieving near-canonical rates with short spacers, significantly reduced when extended	Multi-input logic, spatially programmable circuits, kinetic profiling
Associative activation	Key domains are split and must be assembled by an auxiliary strand to enable displacement	Wide kinetic range; fine and flexible tuning through spatial design	Multi-strand logic circuits, molecular computation, gated sensing
Hierarchical activation	Multi-step activation via hidden toeholds (hairpin/photocage), allosteric toehold, cooperative BM, handhold	Introduces intermediates and delays for temporal control; reduced leakage; non-equilibrium behavior	Complex circuits, conditional biosensing, cascades, dynamic nanodevices
Toehold-free	Driven by entropy, fraying ends, multivalency, mismatched strands, or	Generally slower (lack nucleation) but tunable by multivalency/mismatch/local	Adaptive DNA nanostructures, unconventional

	metal-induced base switching	concentration; avoids explicit-toehold leakage; higher design freedom	computing modules, condition-responsive systems
Enzymatic	Polymerase extension, ligation, cleavage of blockers, helicase unwinding, CRISPR-driven	Bypasses the requirement for a toehold; rapid activation (polymerase/helicase), switch-like control (nuclease/ligase), high specificity (CRISPR); non-equilibrium regulation	Biosensing and diagnostics, synthetic biology circuits, drug delivery
Global factors	Environmental modulation: pH, ionic strength, temperature, crowding agents, small-molecule binders	Broad, reversible, context-dependent impact on duplex stability and exchange rates; often lacks sequence specificity	Generalized rate modulation; enhancing robustness under non-ideal conditions; screening for DNA binders; improving sensing fidelity
Advanced dynamic control	External fuel consumption, transient blocking, feedback loops, in situ generation of nucleic acids, and other non-equilibrium designs layered onto SDRs	Operates out of equilibrium: reactions become autonomous, reversible, and temporally programmable	Life-like dynamic devices, biosensing, therapeutic delivery, autonomous circuits, and smart delivery systems
